# Co-infection with *Anaplasma platys, Bartonella henselae* and *Candidatus* Mycoplasma haematoparvum in a veterinarian

**DOI:** 10.1186/1756-3305-6-103

**Published:** 2013-04-15

**Authors:** Ricardo G Maggi, Patricia E Mascarelli, Lauren N Havenga, Vinny Naidoo, Edward B Breitschwerdt

**Affiliations:** 1 Department of Clinical Sciences, Intracellular Pathogens Research Laboratory, Center for Comparative Medicine and Translational Research, College of Veterinary Medicine, North Carolina State University, 1060 William Moore Drive, Raleigh, NC, 27607, USA; 2 School of Veterinary Medicine, St George’s University, West Indies, Grenada; 3 Biomedical Research Centre, Faculty of Veterinary Science, University of Pretoria, Pretoria, South Africa

**Keywords:** *Bartonella*, *Mycoplasma*, *Anaplasma*, Headache, Migraines, Seizures, Serology, PCR

## Abstract

**Background:**

During a two year period, a 27-year-old female veterinarian experienced migraine headaches, seizures, including status epilepticus, and other neurological and neurocognitive abnormalities. Prior to and during her illness, she had been actively involved in hospital-based work treating domestic animals, primarily cats and dogs, in Grenada and Ireland and anatomical research requiring the dissection of wild animals (including lions, giraffe, rabbits, mongoose, and other animals), mostly in South Africa. The woman reported contact with fleas, ticks, lice, biting flies, mosquitoes, spiders and mites and had also been scratched or bitten by dogs, cats, birds, horses, reptiles, rabbits and rodents. Prior diagnostic testing resulted in findings that were inconclusive or within normal reference ranges and no etiological diagnosis had been obtained to explain the patient’s symptoms.

**Methods:**

PCR assays targeting *Anaplasma* spp. *Bartonella* spp. and hemotopic *Mycoplasma* spp. were used to test patient blood samples. PCR positive amplicons were sequenced directly and compared to GenBank sequences. In addition, *Bartonella* alpha Proteobacteria growth medium (BAPGM) enrichment blood culture was used to facilitate bacterial growth and *Bartonella* spp. serology was performed by indirect fluorescent antibody testing.

**Results:**

*Anaplasma platys, Bartonella henselae* and *Candidatus* Mycoplasma haematoparvum DNA was amplified and sequenced from the woman’s blood, serum or blood culture samples. Her serum was variably seroreactive to several *Bartonella* sp. antigens. Despite symptomatic improvement, six months of doxycycline most likely failed to eliminate the *B. henselae* infection, whereas *A. platys* and *Candidatus* M. haematoparvum DNA was no longer amplified from post-treatment samples.

**Conclusions:**

As is typical of many veterinary professionals, this individual had frequent exposure to arthropod vectors and near daily contact with persistently bacteremic reservoir hosts, including cats, the primary reservoir host for *B. henselae,* and dogs, the presumed primary reservoir host for *A. platys* and *Candidatus* Mycoplasma haematoparvum. Physicians caring for veterinarians should be aware of the occupational zoonotic risks associated with the daily activities of these animal health professionals.

## Background

Many veterinary professionals (veterinarians, veterinary technicians and veterinary support personnel) are occupationally exposed to a spectrum of domestic, production and wild animals, a subset of which can be persistently infected with bacteria, protozoans and viruses. In addition to extensive contact with infected animals and their biological fluids and tissues while performing clinical and necropsy examinations, veterinarians are frequently exposed to arthropod vectors, such as fleas, lice and ticks that infest healthy, sick and dead animals. Also, veterinarians involved in the care of production animals, wildlife or zoological collections have additional environmental exposure to biting flies, chiggers, mosquitoes, spiders and other arthropods while working in terrestrial and marine coastal environments throughout the world. Although it is well recognized that approximately 60% of all human pathogens and 75% of emerging pathogens are zoonotic in nature [1-3], little attention has been focused on the occupational risks associated with the daily professional activities of veterinarians.

Recently, infections with various *Bartonella* species, including *Bartonella henselae*[4-6], *Bartonella koehlerae*[6,7], *Bartonella vinsonii* subsp. *berkhoffii* genotypes I and II [4,6,8,9] and *Candidatus* Bartonella melophagi [10] have been reported among veterinary professionals. Two studies have also supported potential needle stick transmission of *B. vinsonii* subsp. *berkhoffii* and *B. henselae* to veterinarians, respectively [11,12]. In a study in which many of the participants were veterinary professionals [6], *Bartonella* spp. seroreactivity or bacteremia was documented in 49.5% (n = 95) and 23.9% (n = 46) of 192 patients, respectively; however, IFA antibodies were not detected in 30.4% (n = 14) of bacteremic patients. Thus, seronegative *Bartonella* bacteremia is not an uncommon phenomenon. Co-infection with *B. henselae* and *Mycoplasma ovis* was also recently described in a veterinarian, who reported frequent bites or scratches from cats, dogs, rodent pocket pets, and an assortment of wild and zoo animals [13]. On numerous occasions, that veterinarian had traveled for professional activities to Central America and Colombia. Also, while working in Texas, he was exposed to sheep, goats, llamas, camels and had frequent deer contact throughout his career. The exposure history and travel experiences reported by the Texas veterinarian and the veterinarian described in this report are typical of the experiences of many veterinary professionals working around the world throughout their careers.

*Anaplasma platys,* transmitted by the Brown Dog Tick (*Rhipicephalus sanguineus*), is an obligate intracellular rickettsial organism that infects platelets [14,15]. In 1978, this bacterium was first described in the southeastern United States as the cause of canine infectious cyclic thrombocytopenia. Historically, *A. platys* was thought to only infect dogs, however, a recent report from Brazil implicated *A. platys* infection in a cat [16].

In 2004, Sykes and colleagues described a novel hemotropic mycoplasma in the blood of a splenectomized immunocompromised dog with haemic lymphoid neoplasia and proposed the name *Candidatus* Mycoplasma haematoparvum [17]. We now report the medical history for a sick veterinarian from Grenada, who was infected with *A. platys, B. henselae* and *Candidatus* M. haematoparvum.

### Case report

Prior to the onset of her symptoms, a 27-year-old female veterinarian had been actively involved in hospital-based work treating domestic animals, primarily cats and dogs, and anatomical research dissecting wild animals (including lions, giraffe, rabbits, mongoose, and other animals). These activities had occurred in Grenada, Ireland and South Africa. The woman reported contact with fleas, ticks, lice, biting flies, mosquitoes, spiders and mites. She also reported scratches or bites by dogs, cats, birds, horses, reptiles, rabbits and rodents. Beginning in September, 2010, headaches of approximately two weeks duration preceded a fainting episode, photophobia, generalized muscle fasciculations and the onset of tonic-clonic seizures. When hospitalized in South Africa, a CBC and serum chemistry profile were unremarkable and a contrast brain CT revealed no structural abnormalities or evidence of vascular damage. A few days later, encephalitis was diagnosed based on MRI lesions that were considered characteristic for Herpes simplex encephalitis. The patient was admitted to the Intensive Care Unit (ICU), and over the next 10 days was maintained on a sedative, lorazepam (Ativan), anticonvulsants including sodium valproate (Epilim) and phenytoin sodium (Dilantin) which was stopped after an adverse reaction, and pain medications including tramadol hydrochloride (Ultram ER) and paracetamol (Perfalgan), as required. Amoxicillin, cephazolin and doxycycline were also administered for 2 weeks. A 24 hour electroencephalogram (EEG) revealed no electrical abnormalities and CSF analysis was not indicative of inflammation or infection. Electron microscopy of CSF revealed protein fibrils suspected to be associated with “tick-bite fever”. Due to the potential of a transmissible infectious agent, the National Institute of Communicable Diseases (NICD) in South Africa tested for Ebola hemorrhagic fever (*Ebolavirus*), Lyme disease (*Borrelia burgdorferi*), spotted fever group rickettsioses (*Rickettsia africae* and *Rickettsia conorii*), Rift Valley Fever (*Phlebovirus*), Equine Viral Encephalitis (*Arbovirus*), African horse sickness (*Orbivirus*) and Rabies(*Lyssavirus*), all of which were negative. The patient was also treated presumptively because of potential *Streptococcus suis* exposure, as she had case contact a few days prior to hospitalization with a piglet that had cerebral signs and a positive brain culture. When discharged from ICU to the ward, she was treated with sodium valproate and carbamazepine (Tegretol). One month post-discharge, the patient continued to experience tremors, mild seizures, ataxia and memory loss and had left-sided weakness. An analgesic containing paracentamol, codeine phosphate, caffeine, and meprobmate (STILPNE® Capsules) was added to the treatment regimen. During the next two weeks seizure frequency, severity, and duration increased, with seizures becoming more violent and lasting at least 10 minutes. When referred to a neurologist, the patient was monitored on the neurology ward using a three-day camera linked to an EEG, which documented seizures occurring four to seven times a day. As seizures accompanied ringing noises (drip alarms, jiggling bottles on trolleys, etc.), anticonvulsants were discontinued on the premise that seizures were induced by sound hyper-sensitivity and were potentially being accentuated by the medications. Sertraline hydrochloride, a selective serotonin reuptake inhibitor (SSRI) was administered for noise hypersensitivity, post-encephalitic depression, disorientation, and irritability. Results of repeat CBC, serum chemistry panel, EEG and MRI were negative or within reference ranges. The neurologist concluded that the lesion(s) inducing the seizure focus was/were microscopic and deeper than detectable by EEG. While hospitalized, a psychologist recommended further personal counseling, as he felt that the patient needed to come to terms with post-encephalitic seizures and inability to be active and to live the life she experienced before becoming ill. Before the onset of symptoms, the woman was actively involved in windsurfing, diving, surfing, running and sailing and had completed the Dublin marathon less than a year before the onset of her illness.

One month later, headaches continued, migraines were diagnosed, and vertigo had developed especially when in a moving vehicle, however the left sided weakness had improved and repeat memory testing documented substantial improvement in short term memory with residual deficits in math skills. Amitriptyline (Elavil) and clonazepam (Rivotril) were administered for treatment of the migraines and anxiety, respectively. By four months after the onset of illness, the patient associated her seizures with loud noises, bright light or long car rides, all of which she attempted to avoid. One month later, she was again hospitalized due to severe migraines, inability to enunciate words and left-sided weakness. A CBC, serum chemistry panel, and a CT scan were negative or within reference ranges. Sodium valproate was restarted after consultation with a second neurologist. A combination drug containing tramadol hydrochloride and acetaminophen (Tramacet) were added to the treatment regimen for the migraines. When discharged five days later, amitriptyline, sodium valproate, sertraline hydrochloride and Tramacet were continued until August, 2011, at which time the patient elected to discontinue medications against medical advice. One week later, she was hospitalized for debilitating migraines, which persisted for seven days despite administration of multiple medications. Again, there were no MRI abnormalities. She was discharged by her neurologist with instructions to take clobazam (Urbanol), agomelatine (Valdoxin) and amitriptyline for depression and management of the noise hypersensitivity, Tramadol for headaches, and topiramate 100 mg bid (Topamax) as an anticonvulsant and lorazepam (Ativan SL) for emergency seizure control. Between September 2011 and January 2012, these medications were continued and the patient experienced remarkable improvement, with decreased sensitivity to flashing lights, increased tolerance to loud noises, increased energy and improved mental capacities. However in November 2011, she was admitted to the emergency room following the development of joint pain, particularly severe in the knees, left wrist, right elbow and lower back. Standard view radiographs of her left wrist, which was the most severely affected source of pain, revealed no structural abnormalities, CBC values were within reference ranges and an ANA panel, uric acid level and Rheumatoid Factor assay were negative. Initial treatment included intravenously administered dexamethasone, followed by oral steroid maintenance therapy for five days, and Etoricoxib (Arcoxia) 90 mg bid for two weeks. Joint and back pain abated after two weeks.

While in transit from South Africa to Grenada in December 2011, the patient had a seizure in JFK airport in New York, after which she continued to experience severe tonic-clonic seizures while in Grenada. When examined by a neurologist on January 6, 2012, there was a mildly abnormal EEG pattern characterized by bilateral sharp activity, and interpreted as consistent with cerebral irritability in a patient receiving anticonvulsant therapy. Medications now included topiramate (200 mg bid), lamotragine (Lamictal), clonazepam, amitriptyline, agomelatine (Valdoxane) and Tramadol as needed. Over the next two months the seizures decreased in frequency and severity. Concurrently, the woman’s migraines increased in frequency and duration, often lasting 5 days. On March 1st, the patient was airlifted from Grenada to Trinidad in status epilepticus. She remained hospitalized until March 8th, during which time therapy targeted seizures and migraines, but would not have addressed an underlying infection. After discharge, the migraines continued, requiring periodic administration of buprenorphine (Temgesic), which rapidly stopped the migraine within minutes of administration.

Twenty months after the onset of the patient’s illness, one of the authors visited St George’s University to participate in a research collaboration involving canine ehrlichiosis, which is a highly endemic canine tick borne disease on the island of Grenada. After reciting her medical history during a casual conversation, the woman elected to enter an ongoing study regarding *Bartonella* spp. infection in high risk patients, i.e. veterinary professionals (North Carolina State University Institutional Review Board approval IRB 1960–11). Written permission was given to also test for other vector borne organisms.

## Methods

### Serology

For this study, all serum samples were tested by IFA assays using a panel of *Bartonella* antigens. Briefly, antibody responses to *Bartonella henselae* strain Houston I, *B. henselae* strain San Antonio 2, *B. vinsonii* subspecies *berkhoffii* genotype I*, B. vinsonii* subspecies *berkhoffii* genotype II , *B. vinsonii* subspecies *berkhoffii* genotype III, and *B. koehlerae* were tested by IFA as previously described [4-6]. Seropositive samples were defined as having endpoint titers ≥ 1:64 using a twofold scale of 1:16 – 1:8192.

### Molecular testing

*Bartonella* testing was performed using the BAPGM platform, as previously described [4-8]. The BAPGM platform incorporates 4 separate PCR testing time points, each representing a different component of the testing process for each patient sample: 1) and 2) PCR amplification of *Bartonella* spp. following DNA extraction from whole blood and from serum; 3) PCR following BAPGM enrichment of whole blood culture incubated for 7 and 14 days; and 4) PCR from subculture isolates if obtained after subinoculation from the BAPGM flask onto plates containing trypticase soy agar with 10% sheep whole blood that are incubated for 4 weeks. PCR specimen preparation, DNA extraction, and PCR amplification and analysis were performed in three separate rooms with unidirectional work flow to avoid DNA contamination. In addition, BAPGM cultures were processed in a biosafety cabinet with Hepa filtration in a limited access Biosafety Level II laboratory. PCR-negative controls were prepared using 5 μL of DNA from the blood of a healthy dog, and *B. henselae* (Houston 1 strain) at a concentration of 1 genome copy/μL was used as a PCR-positive control during the entire course of this study. To assess for potential contamination during blood sample processing into BAPGM, an un-inoculated BAPGM culture flask was processed simultaneously and in an identical manner with each batch of patient blood and serum samples tested. In addition, molecular testing aiming at amplify *Anaplasma* (16SrRNA and GroEl genes), *Babesia* (18SrRNA), *Ehrlichia* (16SrRNA and GroEl genes), and hemotropic *Mycoplasma* (16SrRNA and RNaseP genes) was performed on DNA extracted from blood and serums samples using primers as previously described [18-21].

## Results and discussion

In April 2012, whole blood and serum sample sets were obtained on Monday, Wednesday, Friday and the following Monday and shipped overnight express to the North Carolina State University, College of Veterinary Medicine, Intracellular Pathogens Research Laboratory (NCSU-CVM-IPRL), for *Bartonella* spp. serology and inoculation into *Bartonella* alpha Proteobacteria growth medium (BAPGM).

The patient was seroreactive to *B. vinsonii* subsp. *berkhoffii* genotype II (titer 1:256) and *B. henselae* antigens (1: 64), but was not seroreactive to *B. vinsonii* subsp. *berkhoffii* genotypes I and III or to *Bartonella koehlerae* antigens at the lowest testing dilution of 1:16. *Bartonella* spp. DNA was not amplified from four blood, four serum, or six enrichment blood culture samples obtained at 7 and 14 days post-incubation and no subculture isolates on blood agar were obtained. For all components of the BAPGM platform (PCR from blood, serum, enrichment cultures at 7 and 14 days, and subcultures), PCR-negative controls remained negative throughout the course of the study. In addition, subcultures of un-inoculated BAPGM medium (culture control) at 7 and 14 days did not yield bacterial growth.

In contrast, by targeting a conserved region of the 16S *rRNA* gene *A. platys* DNA was PCR amplified and sequenced from all four serum and 2 of 4 blood extracted DNA samples. (Table 1) The six amplified sequences were identical to each other and had 99.7% (350/351 bp) homology with *A. platys* (M82801) deposited in GenBank. Similarly, amplification of the *GroEL* gene generated a 450 bp product that was 98.3% similar to *A. platys* GenBank AY008300, and 98.9% (444/446) similar to *A. platys* GenBank accessions AF478129, and AF399916. In addition, *Candidatus* M. haematoparvum DNA was amplified and sequenced from two of the patient’s serum samples using primers targeting a conserved region of the 16S *rRNA* and the *RNaseP* genes [21]. The 16S *rRNA* gene sequences were 99.8% (400/401 bp) similar to GenBank accession GQ129113 and the *RNAseP* sequences were 100% (128/128 bp) similar to Genbank accession AY380803 of *Candidatus* M. haematoparvum*,* respectively. As the authors were unable to identify a research or commercial laboratory that could provide *A. platys* IFA antigen slides, *A. platys* serology was not possible. Also, as cell wall deficient hemotropic *Mycoplasma* species have not been isolated to date, hemoplasma serology was not performed. *Babesia* and *Ehrlichia* genus PCR assays did not result in DNA amplification.

**Table 1:** **Chronological PCR and *Bartonella *** **spp. serology results for a veterinarian infected with *Anaplasma platys, Candidatus *** **Mycoplasma haematoparvum and *Bartonella henselae***

		**PCR**						**Serology**					
		**Bartonella**			**Anaplasma**		**Hemot. *Mycoplasma***	***Bvb I***	***Bvb II***	***Bvb III***	***Bh***	***Bh SA2***	***Bk***
		**ITS**			**GEP**	**GroEl**	**16S rDNA and R** ***NaseP***	**IFA reciprocal titers**					
**Sample Date**	**Sample**	**Original**	**BAPGM**										
			**7 d**	**14 d**									
4/16/2012	Serum	Neg			**A. *platys***	**A. *platys***	Neg	< 16	256	< 16	64	< 16	< 16
	Blood	Neg	Neg	Neg	Neg	Neg	***M. haematoparvum***						
4/18/2012	Serum	Neg			**A. *platys***	Neg	Neg						
	Blood	Neg	Neg	Neg	**A. *platys***	Neg	Neg						
4/20/2012	Serum	Neg			**A. *platys***	**A. *platys***	***M. haematoparvum***						
	Blood	Neg	Neg	Neg	Neg	Neg	Neg						
4/23/2012	Serum	Neg			**A. *platys***	Neg	Neg						
	Blood	Neg	Neg	Neg	**A. *platys***	Neg	Neg						
5/7/2012	Serum	Neg			**A. *platys***	**A. *platys***	**A. *platys***	< 16	64	32	< 16	128	64
	Blood	Neg	Neg	Neg	Neg	**A. *platys***	**A. *platys***						
5/9/2012	Serum	Neg			Neg	Neg	Neg						
	Blood	Neg	Neg	Neg	Neg	Neg	Neg						
5/11/2012	Serum	Neg			Neg	Neg	Neg						
	Blood	Neg	Neg	Neg	Neg	Neg	Neg						
5/14/202	Serum	Neg			**A. *platys***	Neg	Neg						
	Blood	Neg	Neg	***Bh *** **SA2**	Neg	Neg	Neg						
12/10/2012*	Serum	Neg			Neg	Neg	Neg	< 16	256	128	128	128	< 16
	Blood	Neg	Neg	Neg	Neg	Neg	Neg						
12/12/2012*	Serum	Neg			Neg	Neg	Neg						
	Blood	***Bh *** **SA2**	Neg	Neg	Neg	Neg	Neg						
12/14/2012*	Serum	Neg			Neg	Neg	Neg						
	Blood	Neg	Neg	Neg	Neg	Neg	Neg						

*Bh* SA2 = *Bartonella henselae* San Antonio 2 strain, *Bvb* = *Bartonella vinsonii* subsp. *berkhoffii.*

*Bk* = *Bartonella koehlerae,* Hemot. = Hemotropic, N/D = Not determined, * = Post treatment, Neg = Negative.

When the *A. platys* and *Candidatus* M. haematoparvum PCR results became available, an additional set of aseptically obtained blood and serum samples were submitted for repeat *Anaplasma*, *Bartonella*, and hemotropic *Mycoplasma* spp. testing. Four sample sets collected between May 5 and May 14, 2012 were shipped to the NCSU-CVM-IPRL by overnight express carrier. The patient was again seroreactive to *Bartonella* spp. antigens by IFA testing. Table 1*Bartonella henselae* (SA2 strain type) DNA was amplified and sequenced from a 14 day BAPGM enrichment blood culture. *Bartonella* ITS PCR was negative for 4 blood, 4 serum, 4 seven day enrichment blood cultures and 3 of 4 14 day enrichment blood cultures. No subculture agar plate isolates were obtained at any testing time points (April and May 2012). *Anaplasma platys* DNA was again successfully amplified and sequenced from two of the patient’s four serum samples. The *A. platys* DNA sequences were identical to the sequences derived from the April blood and serum samples. *Candidatus* M. haematoparvum DNA was not amplified from the May blood or serum samples.

Following NCSU-CVM-IPRL confirmation of infection with *A. platys, Candidatus* M. haematoparvum, and *B. henselae* the patient returned to South Africa before initiating antimicrobial treatment on July 18, 2012. Based on the longevity of her illness, the attending physician requested a standard echocardiogram, CBC, C-reactive protein and Lyme serology (negative) be repeated. The only hematological abnormality was a mild increase in C-reactive protein. When the echocardiogram revealed slight thickening of the mitral valve, a trans-esophageal echocardiogram was obtained under deep sedation. Thickening of the mitral valve was attributed to age-related myxoedamatous degeneration. Treatment was initiated with doxycycline (100 mg bid) for 6 months. Concurrent administration of azithromycin or rifampicin was not attempted due to concerns that these antibiotics could interfere with the anticonvulsant medications, resulting in patient destabilization. During the first week of doxycycline administration, the patient experienced several days in which severe tonic-clonic seizures of a few minutes duration, occurred up to three times per day. Seizures were followed by disorientation and severe migraines, with the latter only responsive to buprenorfine (Temgesic SL). Following a week of doxycycline treatment, the patient reported less frequent seizures, more clarity in her thoughts. Also, the historical lethargy, which had been a constant symptom since contracting encephalitis in September 2010, had substantially resolved.

Approximately one month before commencing antibiotic treatment, the patient injured her right wrist during a seizure. After a series of radiographs and an MRI scan, a scapholunate ligament tear was confirmed. The MRI also identified minute osteolytic lesions involving the joint surfaces of numerous bones within the wrist along with generalized osteopenia. The scapholunate ligament tear required surgical correction. Within two weeks of surgery, the patient developed complex regional pain syndrome (CRPS), requiring a follow-up bone scintigraphic scan in August 2012 that identified increased uptake in the scaphoid, lunate and pisiform bones of the right wrist and the periarticular joints distal to the right wrist. There was also moderate to intense linear uptake in the right distal ulna. A consulting orthopedic surgeon suggested that the patient’s osteolytic lesions might be similar to lesions reported in immune-compromised patients with *Bartonella* infections.

In December 2012, after 6 months of doxycycline therapy, three aseptically obtained whole blood and serum sample sets were collected in Grenada and shipped overnight express to the NCSU-CVM-IPRL, for *A. platys* PCR, *Bartonella* sp. serology, BAPGM enrichment blood culture/PCR, and *Candidatus* M. haematoparvum PCR, as described above. The patient was seroreactive to *B. vinsonii* subsp. *berkhoffii* genotypes II and III (titers 1:256 and 1:128, respectively) and *B. henselae* antigens (1: 128), but was not seroreactive to *B. vinsonii* subsp. *berkhoffii* genotype I or to *Bartonella koehlerae* antigens at the lowest testing dilution of 1:16. *Anaplasma platys* and *Candidatus* M. haematoparvum DNA was not amplified by the respective PCR assays. *B. henselae* SA2 DNA was amplified and sequenced from one of three blood sample sets, suggesting that the *B. henselae* infection may not have been eliminated by the doxycycline therapy. As *Bartonella* spp. DNA was not amplified from BAPGM enrichment blood cultures, the presence of viable bacteria was not documented.

Clinically, following the six months of doxycycline therapy, the patient was more alert, enjoyed a more active life-style and cognition had greatly improved. However, following periods of overexertion, she continues to develop lethargy, followed by severe migraines, which require treatment with analgesics or bed rest. The patient is being transitioned off of the anti-epileptic medications. Beginning in November 2011, because the patient was experiencing severe insomnia, lamotrogine was tapered and completely withdrawn without an increase in seizure frequency. Topiramate is being tapered gradually until a lowest effective dose is found, or the drug can be withdrawn completely. The patient was advised that the amitriptyline can be stopped once the frequency and intensity of the migraines decrease, whereas valdoxane will be continued until the patient is less sensitive to noise. If topiramate can be tapered, the treatment plan is to add rifampin and continue doxycycyline.

Documentation of co-infection with three vector-borne organisms in the same patient, two of which (*A. platys* and *Candidatus* M. haematoparvum) have not been described in association with human blood-borne infections, represents a medically important observation derived from sequential testing of blood samples provided by this veterinarian. As is typical of many veterinary professionals, this woman had frequent exposure to arthropod vectors and near daily contact with persistently bacteremic reservoir hosts, including cats, the primary reservoir host for *B. henselae,* and dogs, the primary reservoir host for *A. platys*[22,23] and *Candidatus* M. haematoparvum [21]. Based upon serological evidence, this veterinarian may have been exposed to both *B. henselae* and *B. vinsonii* subsp. *berkhoffii.* A previous study from Grenada documented a 19.2% *A. platys* PCR prevalence and an 8.2% *B. vinsonii* subsp. *berkhoffii* seroprevalence among dogs [24]. Those dogs were not tested serologically for *B. henselae* exposure, nor was BAPGM enrichment blood culture/PCR, which increases molecular diagnostic sensitivity [25,26], performed in that study, as was done with blood and serum specimens from this patient. It is obvious from the results summarized in Table 1 that consistent PCR amplification of each of these three organisms from blood, serum or enrichment blood culture samples represents an ongoing challenge for molecular diagnostic laboratories. Presumably, the patient’s *B. henselae* infection was missed when she was first tested in April, 2012, and only one of three BAPGM enrichment cultures documented viable *B. henselae* infection when retested one month later and only after a 14 day BAPGM incubation period. Recently, we reported a statistical increase in the molecular detection or isolation of *Bartonella* spp. when three blood sample sets were tested from a one week collection period, as compared to testing a single blood sample [27]. *Bartonella* detection in blood by PCR and/or following enrichment blood culture remains difficult to achieve, due to the potential for very low numbers of bacteria in the patient’s blood at the time of sample collection and because of the suspected relapsing nature of the bacteremia in immunocompetent individuals [27]. As *B. henselae* SA2 strain DNA was again sequenced from a single blood sample obtained in December 2012, after six months of doxycycline therapy, it seems likely that the woman remained infected, although it is possible that the amplified DNA in September was from dead or nonviable bacteria, whereas growth in enrichment culture in May would reflect the presence of viable blood borne bacteria. Treatment failure seems more likely in this patient, as doxycycline alone is not a consistently effective antibiotic for the elimination of *B. henselae* bacteremia and bacterial DNA does not persist for months in the blood, after the infection has been eliminated [13]. An ongoing limitation of the BAPGM enrichment blood culture platform is the failure to obtain isolates from most patients following subculture at 7 and 14 days of incubation [6].

In addition to treating companion animals and wildlife on three different continents, this veterinarian had the added risk of performing frequent wildlife necropsies in Grenada and South Africa, including lions and mongoose (NCSU-CVM-IPRL, Unpublished data) that could be a source of *B. henselae* exposure. Although NCSU-CVM-IPRL research testing efforts focused on vector-borne organisms of veterinary medical importance, it is possible that this woman was exposed to or infected with other pathogens that contributed to or influenced her clinical course of illness prior to or during the course of this investigation. Therefore, correlation of patient symptoms and disease findings with the microbiological findings reported as a component of this study should be interpreted with caution. However, in association with improved diagnostic testing modalities, co-infections with more than one vector-borne pathogen are being reported frequently in dogs and occasionally in human patients. As cats and dogs are more often exposed to fleas, ticks and other vectors as compared to their human counterparts, co-infections are commonly reported to occur in pet and working dogs with frequent flea and tick infestations [28-31]. These clinical observations have prompted veterinary researchers to study *A. platys* and *E. canis* co-infections in dogs experimentally [32]. Among other examples in the human medical literature, co-infection with *E. chaffeensis* and a spotted fever group *Rickettsia* was reported in a 44-year-old man [33]. Of medical importance to physicians and veterinarians, co-infection with organisms that can potentially persist for months to years complicates a patient’s clinical presentation, can substantially influence the historical progression of illness and can make laboratory diagnosis much more challenging than an acute infection or an infection by a single pathogen. Also, in selected patients, co-infections can influence the choice of therapeutic agents, for example when a patient is infected with a bacterium and a protozoa [34]. Whenever possible, PCR amplification with DNA sequence confirmation, as was used in this study, should be the basis for diagnosis of a co-infection. Because microbial specific genes are targeted in well designed PCR assays, this increasing useful diagnostic approach is applicable to both human and veterinary patient populations. In this study, all PCR amplicons were sequenced to assure specificity. Bacteremia with the two novel organisms for human infection (*A. platys* and *Candidatus* M. haematoparvum) was confirmed by targeting two different genes, whereas DNA sequencing of the highly variable *Bartonella* 16S-23S ITS region was used to confirm infection with a SA2 strain of *B. henselae*.

During the two-year period prior to documentation of *A. platys, B. henselae* and *Candidatus* M. haematoparvum infection in this patient, extensive diagnostic testing was pursued in conjunction with efforts to define the cause of the headaches, seizures and other neurological and neurocognitive abnormalities. Unfortunately, those tests proved to be normal, negative, or inconclusive in the context of identifying an etiological diagnosis. The lack of fever, in conjunction with normal hematological, serum biochemical and cerebrospinal fluid findings and several normal MRI examinations after the initial post-encephalitic MRI diagnosis argued against an ongoing infectious cause of the neurological symptoms in this patient. During the patient’s initial illness, the first consulted neurologist was convinced that the MRI lesions were residual from an unusually virulent case of African tick-bite fever. He also believed that the patient would respond to the standard two weeks of doxycycline used to treat *Rickettsia conorii, Rickettsia africae* and *Coxiella burnetii* infections in South Africa. Unfortunately, this treatment did not prove to be sufficient.

In veterinary medicine, *A. platys* and hemotropic *Mycoplasma* sp. are considered pathogens of low virulence, often documented in association with other infections or other diseases. Most dogs infected with *A. platys* are healthy, but experience a cyclic thrombocytopenia; however, strain variation in pathogenicity has been proposed due to more severe disease attributed to *A. platys* infections in dogs in Europe [14,15]. Despite development of thrombocytopenia, dogs experimentally infected with *A. platys* remained healthy throughout a study [32]. Currently there is also minimal evidence to support an important pathogenic role for hemotropic *Mycoplasma* species infecting dogs [17,21] or people [13]. Hemotropic *Mycoplamsa* infections that are accompanied by disease manifestations occur most often in nutritionally deprived or immunologically compromised animals, such as retroviral infected cats. Infection with *Mycoplasma haemofelis* was reported in an HIV-positive human from Brazil [35].

Recently, veterinary professionals have been identified as a high risk group for *Bartonella* spp. bacteremia [6]. Based upon repeat testing, there was serological and BAPGM enrichment blood culture PCR evidence to support *B. henselae* infection in this veterinarian. Although the pathophysiological mechanisms remain essentially unstudied, headaches, seizures and other neurological signs have been reported in patients with *Bartonella* spp. bacteremia [4-8]. Similar to the patient in this report, there is often no history of fever or hematological, biochemical or cerebrospinal fluid indicators of infection in patients with neurobartonellosis [5,8,36]. In addition, co-infection with *B. henselae* and *Mycoplasma ovis,* a hemotropic *Mycoplasma* sp. that infects sheep, has been reported in a veterinarian with long-standing neurological disease [13]. Of the three organisms infecting this patient, *B. henselae* alone, or in combination with the two other intravascular bacteria may have contributed to the headaches, neurocognitive abnormalities and seizures reported in this patient. The osteolytic bone lesions documented in this patient just prior to initiation of antibiotics are also consistent with lesions that have been increasingly described in immunocompromised HIV-infected patients and in children with cat scratch disease [37,38]. Despite the use of different combinations of anticonvulsant and antidepressant medications, effective control of the headaches and seizures was never achieved until treatment with doxycycline was instituted. Unfortunately, serology and PCR results following 6 months of antibiotic therapy supported the possibility of ongoing *B. henselae* infection.

Nearly two decades ago investigators in Venezuela described inclusions in human platelets ultrastructurally consistent with *A. platys*[39,40]. As those observations predated the use of PCR amplification and DNA sequencing, confirmation that the platelet inclusions were in fact *A. platys* was not possible and no subsequent report of human *A. platys* infection has been published in the English literature. Although vector competence has not been proven, there is substantial epidemiological support for *R. sanguineus* as the vector and the dog as the primary reservoir host for *A. platys*[29] and potentially *M. haematoparvum*[21] and *B. vinsonii* subsp. *berkhoffii*[41]*.* Dogs throughout tropical and subtropical regions of the world are frequently infested with *R. sanguineus,* commonly referred to as the “brown dog tick” or “kennel tick” because all three life stages (larvae, nymph and adult) prefer to feed on dogs and these ticks are frequently found in kennel environments, veterinary hospitals and homes in tropical and subtropical regions of the world. Grenada, located approximately 100 miles from Venezuela, shares very similar rural and urban ecosystems, each of which supports frequent and severe *R. sanguineus* infestations in dogs. In fact, *R. sanguineus* is the only tick known to infest dogs on the island of Grenada and is a commonly encountered tick on dogs in South Africa. Although an important vector for transmission of *Babesia canis* and *Ehrlichia canis* throughout the world, historically, the human medical importance of this tick has been underappreciated. Recently, *R. sanguineus* has been implicated in the transmission of *Rickettsia rickettsii* on Indian reservations in Arizona, resulting in fatal cases of Rocky Mountain spotted fever [42]. *R. sanguineus* is also the vector for *Rickettsia conorii,* the cause of Mediterranean spotted fever in dogs and people in southern Europe and northern Africa. Recently, persistent *R. conorii* bacteremia has been demonstrated experimentally in dogs infected by tick (*R. sanguineus*) attachment [43]. Although the timing and mode(s) of infection for this patient will remain unknown, the importance of *R. sanguineus* as a source of *A. platys* and *Candidatus* M. haematoparvum for dogs and people deserves additional research consideration. Similarly, fleas, the primary vector for transmission of *B. henselae* and likely other *Bartonella* spp. among cats and dogs, are currently underappreciated as a source of zoonotic bartonellosis among animals and human patients [44].

## Conclusion

As is typical of many veterinary professionals, this individual had frequent exposure to arthropod vectors and near daily contact with persistently bacteremic reservoir hosts, including cats, the primary reservoir host for *B. henselae,* and dogs, the presumed primary reservoir host for *A. platys* and *Candidatus* Mycoplasma haematoparvum. Due to frequent contact with ticks and fleas, and the animals that harbor intravascular vector-borne pathogens for months to years, veterinary professionals should use personal protective measures, such as gloves, hand washing and optimal restraint to avoid bites and scratches. Rapid kill spray products should be used routinely to eliminate fleas and ticks from animals that are being examined at necropsy or cared for by veterinary professionals.

More importantly, physicians caring for veterinary professionals should be aware of the occupational zoonotic risks associated with the daily activities of animal health professionals.

## Consent

Written informed consent was obtained from the patient for publication of this report and any accompanying images.

## Abbreviations

(NICD): National Institute of Communicable Diseases; (CRPS): Complex Regional Pain Syndrome; (BAPGM): *Bartonella* Alpha Proteobacteria Growth Medium; (NCSU-CVM-IPRL): North Carolina State University College of Veterinary: Medicine, Intracellular Pathogens Research raboratory.

## Competing interests

In conjunction with Dr. Sushama Sontakke and North Carolina State University, Dr. Breitschwerdt holds U.S. Patent No. 7,115,385; Media and Methods for cultivation of microorganisms, which was issued October 3, 2006. He is the chief scientific officer for Galaxy Diagnostics, a company that provides diagnostic testing for the detection of *Bartonella* species infection in animals and human patients. Dr. Ricardo Maggi has lead research efforts to optimize the BAPGM platform and is the Scientific Technical Advisor for Galaxy Diagnostics. All other authors have no potential competing interest.

## Authors’ contributions

RM and PM performed the BAPGM enrichment blood culture, PCR testing of the patient’s samples, DNA sequencing and alignments, and helped generate the first draft of the manuscript. LNH and VN assisted in sample acquisition, provided medical information and helped write the case history. EB coordinated various aspects of the investigation and helped to draft the final manuscript. All authors read and approved the manuscript.
